# Suicidality in a 27-year-old Male with Obsessive Compulsive Disorder

**DOI:** 10.7759/cureus.3804

**Published:** 2018-12-31

**Authors:** Brittany M Ross, Jessica D Lee, Eduardo D Espiridion

**Affiliations:** 1 Osteopathy, West Virginia School of Osteopathic Medicine, Lewisburg, USA; 2 Psychiatry, Frederick Memorial Hospital, Frederick, USA

**Keywords:** suicidality, exposure and response prevention, obsessive compulsive disorder, cognitive behavioral therapy, ocd pharmacotherapy, refractory ocd

## Abstract

This is a case report of a 27-year-old male with obsessive compulsive disorder (OCD) and anxiety who presented to a community hospital with suicidality. OCD is a rare psychiatric disorder characterized by recurrent and intrusive thoughts or obsessions and/or repetitive behaviors aimed at alleviating these thoughts known as compulsions. Management of this condition includes comprehensive evaluation of comorbidities and suicidality along with pharmacotherapy and a specific form of cognitive behavioral therapy (CBT) called exposure and response prevention (EX/RP or ERP). This unique case report highlights the necessity of a thorough and individualized approach to treatment for each patient in order to maximize the outcomes of care.

## Introduction

Obsessive compulsive disorder (OCD) is a chronic disorder which can significantly impair functioning in patients and may be associated with comorbid psychiatric conditions which can lead to suicide [1, 2]. OCD is characterized by recurrent unwanted thoughts and urges to complete behaviors in response to these thoughts. These behaviors are time-consuming, taking up at least one hour a day. This disease can wax and wane over time and patients may present years after the onset of symptoms [3]. The specific diagnostic criteria of OCD can be found in the Diagnostic and Statistical Manual of Mental Disorders, 5th Edition (DSM-5) [4]. Patients with OCD can have a diminished quality of life and in one study nearly 82% of people reported impairment in home, work, relationships or social life [4, 5]. OCD has no gender predominance although males have earlier age of onset [4]. The prevalence has been estimated at 2.3% with a mean age of onset at 16.9-19.5 years [5, 6]. Others have estimated a prevalence between 1 and 3%, however, it is possible this is an underestimation given that not all patients seek treatment for their condition [2, 3]. Genetic and environmental factors are said to play a role in the development of this condition. Pathophysiology of the disorder is not proven but posited theories include changes in neurobiology of specific areas such as the orbitofrontal cortex (OFC), anterior cingulate cortex (ACC), basal ganglia and thalamic structures, autoimmune disease, as well as abnormal serotonin signaling [3, 7, 8].

Major depressive disorder (MDD) is the most commonly reported comorbidity of OCD [1, 2, 5, 6]. MDD has been estimated to have a lifetime prevalence in 40-51% of patients with OCD [5, 6]. Comorbid MDD as well as severity of OCD symptoms, severity of depression, anxiety, feelings of hopelessness and past history of suicidality all have shown to be predictors of suicidality in patients with OCD [9]. In general, half of the individuals with OCD have suicidal thoughts at some point and even up to one-quarter attempts suicide. Additional common comorbidities include some type of anxiety disorder (76%) including generalized anxiety disorder (GAD), panic disorder, social anxiety disorder, depressive or bipolar disorder (63%) with major depressive disorder accounting for 41%. Patients can also present with obsessive compulsive personality disorder (23-32%), history of Tic disorder (30%), and others that are more common in patients with OCD such as trichotillomania, body dysmorphic disorder, hoarding disorder, skin picking disorder, oppositional defiant disorder [4].

Treatment depends on severity of symptoms but patients should be initially managed with psychotherapy as well as pharmacologic treatments. First line therapy is cognitive behavioral therapy (CBT) and pharmacotherapy, beginning with a selective serotonin reuptake inhibitor (SSRI) [3]. Care should be taken to address comorbid conditions in patients with OCD as these can affect the quality of life and prognosis.

## Case presentation

A 27-year-old Caucasian male presented voluntarily to the emergency room of a community hospital with intensifying obsessive-compulsive symptoms and the onset of suicidal ideation. He had been experiencing worsening fear of contamination for approximately one week after a nocturnal emission while camping which led to the feeling that he was unable to clean himself properly. Throughout this past week, he isolated himself at home and was not going to work. His depression was worsening and he reported dysphoria, anxiety, anhedonia, low energy, low motivation, decreased social interest and poor sleep. He stated he had suicidal ideations which were fleeting and denied any intention or plan. He denied hallucinations, delusions, symptoms of mania or hypomania, use of alcohol or illicit substances.

His past medical history is non-contributory. He has a past history of one psychiatric hospitalization. He denies any history of suicidal behavior, history of physically or sexually aggressive behavior and denies any history of drug abuse. Both of his parents have a history of anxiety. He grew up in Maryland and is a college graduate. He lives with his mother and stepfather and works as an editorial assistant. He denies any history of physical or sexual abuse but reports “experimenting” with his stepbrother in middle school.

The patient remembers his anxiety beginning around age 14. It started with trichotillomania, hair pulling, specifically from the back of his scalp, which alleviated anxiety for him. Around age 15, he developed a fear of contamination. This obsession was relieved temporarily with compulsive hand washing. He was managed outpatient for most of his life with the exception of one prior psychiatric hospitalization until this most recent episode following a nocturnal emission while camping. His depression developed secondarily to learned helplessness against his OCD.

Upon admission in the emergency room (ER), the patient’s vitals were taken and labs were drawn. Vital signs were all normal, complete blood count and basic metabolic panel were within normal limits. Urine toxicology screen was negative, and blood alcohol level was <10. The patient reported no additional symptoms other than the presenting complaint upon review of symptoms.

Mental status examination demonstrated a well-groomed, casually dressed patient who was pleasant, cooperative and maintained appropriate eye contact. His speech was of normal rate, rhythm and volume and his affect was full range. His mood was anxious and thought process was logical and goal oriented. His thought content was notable for fleeting suicidal ideation without intention or plan. He denied violent ideations, auditory or visual hallucinations and delusions. His attention span, memory and concentration were grossly intact. His insight, judgement and impulse control were all fair. He was alert and oriented with an adequate fund of knowledge.

Our patient was admitted to the Behavioral Health Unit and was restarted on Risperdal (risperidone) 0.5 mg and Zoloft (sertraline) 200 mg by mouth every evening. We continued monitoring his progress for the next few days. He continued to improve on medications with his compulsive hand washing decreasing from 30 times a day to 10-15 times a day. He participated in group activities and reported no suicidal ideations while on the unit. He was released with the intention of following up with a therapist who was trained in exposure and response prevention (ERP) and following up with psychiatrist who could manage his medications in the community.

## Discussion

The exact pathophysiology of OCD has not been elucidated but there are multiple theories of the evolution of this condition in patients. Treatment with SSRIs results in improvement in some patients with OCD suggesting that abnormal serotonin signaling does play a role in this disease. Mavrogiorgou posits low serotonergic neurotransmission leading to hyperactivity in the orbitofrontal cortex as a potential factor in the etiology of OCD [8]. Serotonin is not the only neurotransmitter implicated in the disease process as patients show improvement in symptomatology while taking medications which also modulate dopamine, norepinephrine and glutamate [3, 7].

Specific brain regions and circuits have been implicated in the disease process. The cortico-striato-thalamo-cortical circuit dysfunction has been linked to OCD. The theory is that there is an imbalance favoring striato-thalamic inhibition leading to hyperactivity in the OFC and ACC. Positron emission tomography (PET) scans have shown increases in glucose metabolism in patients with OCD compared to controls in the thalamus, caudate nucleus and orbitofrontal cortex. This suggests that there are metabolic differences in people suffering from OCD and that treatment directed at these areas may be beneficial [2].

Genetics have been implicated in OCD as well. Presence of a first degree relative with OCD increases the risk to four-fold of that of the population [3]. Specific genes encoding glutamate transporters, such as SLC1A1 and EAAC-1, have been implicated in OCD [7]. Infectious conditions also have a possible connection as OCD symptoms have been seen in children after streptococcal infection (pediatric autoimmune neuropsychiatric disorders associated with streptococcal infections or PANDAS) and after infection with Borna disease virus (BDV). It is thought that immune mechanisms such as cytokine response to BDV increase glutamate activity. Rotge et al. propose a theory demonstrating that the combination of genetic susceptibility and infectious factors leading to increased glutamate neurotransmission can ultimately cause cognitive changes which manifest as OCD [7]. Our patient had a family history of anxiety in general, but not OCD in particular. It is possible that these conditions are linked genetically or that their incidence in his parents increased his susceptibility to developing OCD. He did not report the onset of the symptoms after an infection such as PANDAS as some parents of children with OCD do.

Treatment for OCD consists of pharmacotherapy, psychotherapy and in some cases of refractory OCD, surgical therapy. Pharmacotherapy typically begins with SSRIs such as Celexa (citalopram), Lexapro (escitalopram), Prozac (fluoxetine), Luvox (fluvoxamine), Paxil (paroxetine), and Zoloft (sertraline). SSRIs increase the amount of time serotonin remains the synapse by inhibiting reuptake to propagate its effects on the postsynaptic neuron. Therapeutic doses often need to be higher in OCD than depression. Anafranil (clomipramine) is a tricyclic antidepressant (TCA) typically used after trying and failing SSRIs [3]. It is thought that TCAs modulate norepinephrine as well as serotonin in the brain and may be more effective than SSRIs, however, adverse effects include cardiac arrhythmias, convulsions, and coma. Buspar (buspirone) is another medical option which works on serotonin and dopamine receptors in the brain. Not all patients respond to SSRIs alone and atypical antipsychotics such as Risperdal (risperidone) or Zyprexa (olanzapine) are indicated for augmentation of management with SSRIs. These medications work by blocking dopamine in the brain and may have fewer extrapyramidal side effects than their older counterparts, the typical antipsychotics [3]. Some benefits have been reported with antipsychotic medications augmentation although this is controversial [10]. Our patient had tried multiple medications in the past and was currently taking both an SSRI and an atypical antipsychotic medication, however, he had deteriorated in the community following the episode of nocturnal emission where his OCD worsened, suggesting that the medical management of his condition was not satisfactory. Glutamatergic medications including Namemba (memantine), Mucomyst (N-acetylcysteine), and Rilutek (riluzole) have also been used off-label for the treatment of OCD [3].

Many studies have suggested that OCD is associated with high-risk suicidal behavior. Studies have linked suicidality with the obsessive dimension of OCD to rather than the compulsive dimension [11]. These obsessions can build up leading to a catastrophic level of anxiety in patients and triggering suicidality in many of them [12]. In addition, a case study of treatment-resistant OCD patient has also shown an increase in suicide risk [13]. Thus the severity of OCD can then be a predictor of suicidality in patients. Our patient was open about his suicidal ideation when he presented to the ER. Not all patients will be as forthcoming and it is imperative that as physicians we ask these questions of our patients because researchers have reported a lifetime suicide attempt rate of 14.2% and lifetime suicidal ideation at 44.1% [14]. These statistics are crucial to have in mind as we treat our patients. While many of the treatments are focused on symptomatic improvement, these suicidal ideations can be overlooked if not assessed directly. In fact, a past history of suicidality as well as family history of suicidality has been shown to be predictors of suicidality, increasing the risk by 78% [14]. Thus, asking direct questions including present or past suicidal ideations, attempts, and family history may help to prevent deaths in patients with OCD.

We also have to keep in mind that the obsession itself can be directly related to suicide. OCD with suicidal obsessions has been reported, increasing the need to be able to differentiate between suicidal obsession with suicidal ideations for accurate diagnosis [15]. However, the treatment again should focus on the distress and anxiety caused by the obsession. Therefore, targeting the anxiety-provoking obsessions and concomitant depression may be crucial in preventing suicides in individuals with OCD.

Beyond the sole diagnosis of OCD, comorbidities associated with OCD leads to higher suicidal behavior. The most common comorbid psychiatric diagnoses are depression, anxiety, bipolar affective disorder, and attention deficit hyperactivity disorder [15]. Patients with OCD without any psychiatric comorbidities have significantly increased suicide attempts compared to those without any mental disorder [16]. More alarmingly, patients with OCD with psychiatric comorbidities have even higher lifetime suicide attempts than patients with any other mental disorder [16]. Knowing the possibility of comorbidities with OCD and its association with alarming risk of suicide attempts, the evaluation for more comprehensive management of OCD, therefore a careful insight to patients’ comorbidities, is essential.

Providers should be mindful of the increased risk of suicidal ideation and attempt in their patients with OCD. Identifying these risks can help with prevention and treatment modalities and CBT could also be effective in reducing suicidality as well as treating comorbid mood disorders such as bipolar disorder or major depression [14]. CBT is a form of psychotherapy in which the therapist engages with the patient to increase awareness of negative thinking or behaviors in order to address an issue it/they may be causing. Exposure and response prevention (EX/RP or ERP) is a form of CBT where the goal is to decrease anxiety in relation to triggers through prolonged exposure to the trigger with abstinence from the compulsive behavior tied to it [3]. ERP can help patients separate their obsessions and compulsions. Patients are exposed to triggers and then told to resist compulsions. Participants in one study demonstrated a statistically significant improvement of 56% of patients with OCD showing >25% reduction in symptoms as measured by the Yale-Brown Obsessive Compulsive Scale (Y-BOCS). Patients also reported improved quality of life and social functioning [10]. The degree of ERP benefits has been shown to correlate with patient adherence to treatment. Patients must be willing to confront their fears or obsessions and choose to refrain from performing compulsions or rituals and higher adherence led to lower symptom scores on the Y-BOCS. These objective ratings can also be used to determine the need for continued therapy [17]. Our patient was set up with a provider in the community who incorporated ERP in her CBT sessions. He is a good candidate for this method because he is motivated and therefore likely to remain compliant and have a better chance of success with the program, as measured by lower symptom scores on Y-BOCS and improved quality of life in the aforementioned study completed by Wheaton et al.. Another form of promising CBT in the management of OCD is acceptance and commitment therapy (ACT). ACT works to minimize experiential avoidance which is aversion to situations which may trigger obsessions and changing patients coping strategies thus allowing patients to be more open to experiences. It showed statistically significant improvements in patients in the ACT treatment group with 47.3% reduction in Y-BOCS scores [18].

When all medical and psychotherapeutic options have been exhausted with little or no improvement in OCD symptoms and quality of life, surgery can be considered. Neuroimaging studies have shown the orbito-fronto-striato-thalamocortical fibers, ventral striatum, anterior internal capsule, nucleus accumbens, and the subthalamic nucleus to have increased activity in patients with OCD, making these areas neuroanatomical targets [19]. Deep brain stimulation (DBS) is a reversible and titratable therapy that delivers a current to a specific location. While the exact downstream effects of DBS are not quite understood, many nuclear imaging techniques have shown decreased prefrontal and orbitofrontal cortical metabolism during therapeutic OCD DBS [20]. These effects are similar to metabolic changes observed during pharmacotherapy or behavioral treatment, allowing DBS to be an effective treatment [20]. Another form of therapy is ablative procedures that use radiofrequency to destroy a specific location in the brain. Ablation can either be accomplished by craniotomy or radiosurgical procedure such as gamma knife. Although ablation is permanent and cannot be reversed, DBS has its own disadvantage of requiring adjustment of the implant over time and risk of intracerebral hemorrhage [19]. Both procedures are stereotactic and have a high degree of accuracy. Results are typically seen during the weeks to months after the procedure with reported subjective improvement ranging from 60 to 70% and objective measures showing improvement of 30-45% [2].

## Conclusions

The goal of our inpatient unit is crisis aversion and patient stabilization prior to release back into the community. Our patient presented voluntarily to the emergency room with worsening OCD symptoms and suicidal ideation and while on the unit our patient participated in group therapy and interacted positively with staff and other patients. We restarted his prior medications and he was released to the community with an appointment with a new provider trained in ERP. A thorough assessment of suicidal ideation and behavior is critical in patients with OCD. As these risks increase more dramatically with comorbidities, careful evaluation of other psychiatric conditions will also prove to be important. Our patient had OCD and a depressive disorder that stemmed from his condition, placing him at higher risk of suicidal ideation and attempt. Management of OCD should focus not just on the obsessions and compulsions but also depression, hopelessness and anxiety that may be due to obsession itself, comorbidities or the persistent nature of OCD. Comprehensive management of OCD is necessary as no one therapy option has shown to work universally. With a thorough approach to each patient and the different qualities and symptoms that may accompany their OCD, we can work toward identifying and minimizing the characteristics of a person’s disease. This case demonstrates the necessity for complete and accessible care for patients suffering from OCD and what we can do to ameliorate the lives of those who need help.
